# Potential Limitations in the Clinical Adoption of 3-GEP Pigmented Lesion Assay for Melanoma Triage by Dermatologists and Advanced Practice Practitioners

**DOI:** 10.7759/cureus.31914

**Published:** 2022-11-26

**Authors:** Joanna Ludzik, Claudia Lee, Alexander Witkowski

**Affiliations:** 1 Dermatology, Oregon Health & Science University, Portland, USA; 2 Dermatology, University of California Riverside School of Medicine, Riverside, USA

**Keywords:** tert promoter, prame, linc, 3-gep, dermtech, pigmented lesion assay, melanoma, gene expression profile

## Abstract

Background

A pigmented lesion assay (PLA) is used to non-invasively detect the presence of three genes associated with melanoma (LINC00518, PRAME, and TERT) using adhesive patch testing and has the potential to reduce unnecessary biopsies. However, few studies have evaluated the clinical applicability of PLA testing and its potential limitations in real-world practice. We aim to identify possible barriers that inhibit the clinical utility of PLA testing by dermatologists.

Methods

Retrospective case-control study analyzing the PLA testing by two pigmented-lesion specialists that underwent PLA testing as part of clinical management. Data was collected from April 2021 to April 2022 from an academic tertiary-level center.

Results

The total cohort consists of 472 lesions. Genetic analysis failure for LINC00518 and PRAME occurred in 12.5% of cases and in 70.9% of cases for TERT. In 38.5% of cases, PLA results were discrepant with histopathology. The additional time associated with PLA use independent from the patient’s visit was 15 min on average.

Conclusion

The high proportion of non-actionable results and discrepant cases highlights potential barriers to the widespread adoption of PLA testing. The high proportion of genetic analysis failure seen for TERT and limited influence on the proposed risk suggests TERT does not offer significant clinical value.

## Introduction

As molecular technology in dermatology advances, many emerging in-vivo genetic tools are being increasingly utilized in daily practice. The gene expression profile pigmented lesion assay (PLA) is a noninvasive tape-stripping test based on the expression of three genes (LINC00518, PRAME, and TERT) in the stratum corneum, intended to inform whether a cutaneous lesion is concerning for melanoma [1]. This sampling procedure is painless and may reduce unnecessary surgical biopsies, which is especially useful in cases involving cosmetically sensitive areas or numerous atypical nevi. The test has been validated, reporting high negative predictive values >99% and sensitivity (91-97%) [2-3], essentially claiming to be an effective rule-out method for melanoma. The use of PLA has increased in the past year and may be useful in guiding a dermatology provider's decision to biopsy in challenging cases. However, studies investigating the clinical utility of this test by providers outside of controlled research settings are lacking [4] and possible barriers to its widespread adoption are still unknown.

## Materials and methods

In this large, retrospective case-control study analyzing the use of PLA by multiple pigmented-lesion experts in a highly specialized academic setting at the Oregon Health and Sciences University in Portland, Oregon, conducted from April 2021 to April 2022, we identify limiting factors that may potentially inhibit the clinical utility of the test. Physical biopsies were performed for all PLA (+) cases and for PLA(-) patients with high clinical or dermoscopic concerns determined by the practicing provider. Data, including patient characteristics, histopathology, and PLA results were extracted from electronic medical records of a single-center academic hospital, serving patients with low and high risk for skin cancer. In addition, we tracked feasibility metrics such as time for test acquisition.

## Results

The test was performed on a total of 472 clinically and dermoscopically equivocal pigmented skin lesions. Fifty-nine (12.5%) cases failed from a technical point of view “Quality not sufficient” (QNS) indicating genetic analysis failure for all three mutations. Of the remaining conclusive results, 50 (11.8%) reported a positive PLA test result, indicating the presence of one or more molecular mutations. LINC00518 (n=45, 90%) was the most common mutation detected in positive PLA results followed by PRAME (n=33, 66%); both were present in 24 (48%) of positive cases. When at least LINC or PRAME were detected or confirmed absent, TERT received a QNS result in 300 (70.9%) cases and was detected 13 (11.5%) times. Interestingly, of the 373 reported negative PLA results, which supposedly indicates no presence of mutation expression, TERT was detected in four (1.1%) instances. Table 1 details the distribution of each marker and biopsy results and the corresponding PLA results. A total of 91 biopsies were performed, and in 35 (38.5%) cases the PLA result was discordant with histopathology (PLA (-) with concerning histopathology or PLA (+) with benign histopathology and absence of atypical cytologic features). Concerning histopathology is defined as a diagnosis that may warrant additional treatment or removal. The approximated time for the additional care associated with PLA testing independent of the patient’s visit including acquisition, documentation, submission, and follow-up with results ranged from 10-25 minutes per case, averaging 15 minutes overall.

**Table 1:** Distribution of genetic markers and biopsy results amongst the corresponding PLA results PLA: Pigmented lesion assay

		PLA (+) n=50	PLA (-) n=373
Genetic Markers	LINC00518	45	0
	PRAME	33	0
	TERT	13	4
	LINC00518 + PRAME	24	0
	LINC00518 + PRAME + TERT	5	0
Total Biopsies n=91			
	Positive Pathology n=58	37	21
	Melanoma n=19	14	5
	Atypical Melanocytic nevus n=36	21	15
	Non-melanoma skin cancer n=3	2	1
	Benign Pathology n=33	14	19

## Discussion

Our findings highlight the barriers we faced in applying PLA test results confidently in patient management including a high proportion of non-actionable results and discrepancies between PLA and histopathology in cases that were biopsied (n = 35, 38.5%). We received a considerable amount of overall QNS results (12.5%), where the total amount of extracted RNA was less than required to run any of the 2-GEP markers (LINC00518 and PRAME), which is comparable to the 14% QNS rate reported in one validation study [5]. In this event, the patient was invited back for a second test acquisition attempt in a virtual setting or physical biopsy in-person upon patient request and resulted in an additional 15 minutes spent with the patient in these cases. In five instances (3 AW, 2 JL) where the patient was invited to retest, the PLA result was QNS a second time and the patient ultimately received a biopsy. Additionally, the PLA recently incorporated TERT, a high-risk driver mutation key in early-stage melanoma [1], as an add-on DNA-based molecular assay. In a study including 622 samples, Ferris et al. reported that the addition of TERT in a combined test (LINC00518, PRAME, and TERT) elevated the sensitivity from 91% to 97% and negative predictive value from 99.0% to 99.6% [3,6], however, this study likely did not account for the striking >70% QNS rate for TERT collection which may lend way to overestimation. The high rate of QNS for TERT seen suggests that the marketed 3-GEP PLA test is more appropriately a 2-GEP PLA test (LINC00518 and PRAME) the majority of the time (70.9%). Efforts towards improving the data collection for TERT would enhance the clinical utility of this genomic marker. Furthermore, select cases demonstrated TERT presence in PLA negative results (n=4, 1.1%), which creates additional confusion for providers and questions regarding the utility TERT offers this test.

Other factors that may inhibit the widespread use of PLA testing include limited insurance coverage and inability for the dermatology provider to bill directly for the test, resulting in considerable time spent by the provider and/or staff to complete all processes associated with obtaining a final and actionable test result. Since the test manufacturer contracts directly with insurance payers and the average collection reimbursement per test is 2-3 times that of a physical biopsy to rule out melanoma [7], evaluation of the cost-benefit of this test in widespread use would be prudent. Future multicentered studies evaluating the use of PLA tests in various clinical settings by providers with varying expertise in pigmented lesions are needed to understand the obstacles preventing diffuse clinical implementation and potential overuse when a decision to perform the test is based on ABCD naked-eye criteria alone versus well-adopted standards for dermatology provider screening with dermoscopy.

## Conclusions

The novel non-invasive PLA test for melanoma that utilizes adhesive tape-stripping techniques and gene expression profiling may be a promising, inexpensive solution for reducing unnecessary biopsies and optimizing triage of pigmented lesions. However, studies evaluating the clinical utility, and possible limitations of these tests in a real-world setting are scarce.

Our findings highlight the barriers we faced as dermatologists in applying PLA test results confidently in patient management including a high proportion of non-actionable results and discrepancies between PLA and histopathology in cases that were biopsied. Other factors including cost-effectiveness and time for test acquisition may contribute to provider hesitation with clinical adoption of these tests. As the use of these tests increases, it becomes crucial to highlight the possible limitations in the true clinical utility of PLA testing for melanoma to prevent over-reliance and subsequent mismanagement.
